# Social and dental status along the life course and oral health impacts in adolescents: a population-based birth cohort

**DOI:** 10.1186/1477-7525-7-95

**Published:** 2009-11-22

**Authors:** Karen G Peres, Marco A Peres, Cora LP Araujo, Ana MB Menezes, Pedro C Hallal

**Affiliations:** 1 Research Group in Public Health Dentistry Post-Graduate Program in Public Health, Federal University of Santa Catarina, Florianópolis, Brazil; 2 Post-Graduate Program in Epidemiology, Federal University of Pelotas, Pelotas, Brazil

## Abstract

**Background:**

Harmful social conditions in early life might predispose individuals to dental status which in turn may impact on adolescents' quality of life.

**Aims:**

To estimate the prevalence of oral health impacts among 12 yr-old Brazilian adolescents (*n *= 359) and its association with life course socioeconomic variables, dental status and dental services utilization in a population-based birth cohort in Southern Brazil.

**Methods:**

Exploratory variables were collected at birth, at 6 and 12 yr of age. The Oral Impacts on Daily Performances index (OIDP) was collected in adolescence and it was analyzed as a ranked outcome (OIDP from 0 to 9). Unadjusted and adjusted multivariable Poisson regression with robust variance was performed guided by a theoretical determination model.

**Results:**

The response rate was of 94.4% (*n *= 339). The prevalence of OIDP = 1 was 30.1% (CI95%25.2;35.0) and OIDP ≥ 2 was 28.0% (CI95%23.2;32.8). The most common daily activity affected was eating (44.8%), follow by cleaning the mouth and smiling (15.6%, and 15.0%, respectively). In the final model mother schooling and mother employment status in early cohort participant's life were associated with OIDP in adolescence. As higher untreated dental caries at age 6 and 12 years, and the presence of dental pain, gingival bleeding and incisal crowing in adolescence as higher the OIDP score. On the other hand, dental fluorosis was associated with low OIDP score.

**Conclusion:**

Our findings highlight the importance of adolescent's early life social environmental as mother schooling and mother employment status and the early and later dental status on the adolescent's quality of life regardless family income and use of dental services.

## Introduction

Most clinical and epidemiological studies on oral heath have used clinical parameters as a strategy to evaluate health conditions. However, those parameters only evaluate the physical conditions based on judgments established by professionals - normative assessment - minimizing the psychosocial consequences of the oral conditions [1]. Ideally, the way how individuals perceive and evaluate their health, their symptoms, and consequently their treatment needs, should be included in health surveys. Once the shortcoming of the disease-oriented or biomedical approach has been recognized, the researchers can investigate the impact resulting from the oral health clinical conditions on the quality of life [2].

A variety of sociodental indicators have been developed and used to overcome the normative assessment, with contributions from psychology, sociology, economics, operational research, and biostatistics [2-4]. Some studies have used general questionnaires to measure oral health impacts in children, such as *Oral Impacts on Daily Performance *(OIDP) index for adults [5,6], while other research use specific questionnaire for children [7]. In spite of an increasing number of investigations on the association of dental status with the quality of life in children and adolescents, most of these have addressed specific diseases or conditions, such as orthodontic treatment need [7-9] and dental pain [10,11]. Moreover, when several dental status were simultaneously investigated, we could not identify any strategy to measure the role of confounders, such as multivariable analysis [12].

To date, we found only cross-sectional studies which investigated oral health impacts in children and adolescents [5-9], and are unaware of any population-based study in adolescents that uses a prospective study design. This is of concern because a theory formulated by Barker [13] proposes that there is a critical period of development in early life during which exposures to insults have long-term effects on later health. Moreover, the intensity and duration of exposure to unfavourable or favourable physical and social environments throughout life affects health status in a "dose-response" relationship; it has been termed the "accumulation of risk" hypothesis [14].

From a life course perspective, it can be hypothesized that children from families with low socio-economic conditions in early life may have less access to (and use of) dental services and a variety of oral hygiene items, and may be more likely to develop harmful oral health behaviours later in life [15]. These might predispose individuals to dental status such as dental caries, gingival bleeding, dental pain, malocclusion in adolescence which in turn may impact on adolescents' quality of life.

The aims of this study were to estimate oral health impacts among 12-yr-old Brazilian adolescents and its association with life course socioeconomic variables, dental status and dental services utilization in a population-based birth cohort in Southern Brazil.

## Methods

The study was carried out in Pelotas, a city located in the extreme South of Brazil, close to the border with Uruguay. In 2000, it had a population of 323,158. Pelotas has been water fluoridated since 1961, and about 90% of the city's households are covered.

### The Pelotas' 1993 birth cohort study

The Pelotas' 1993 birth cohort study (*n *= 5,249) was developed mainly to evaluate the trends in maternal and child health indicators through a comparison with results of the early 1982 Pelotas birth cohort study, and to assess the associations between early life variables and later outcomes. All the five maternity hospitals in Pelotas were visited daily during 1993 [15]. The questionnaire applied to the mothers at the maternity hospital included questions about social and economic conditions, demography, pregnancy, behavior, health care, and morbidity. The children were weighed, measured, and examined at birth by a team of doctors and medical students. The sub-samples of the cohort were visited at 1, 3, 6, 12 months, and later, at 4 and 11 yr of age. The home visits included questionnaires administered to mother's and children's anthropometric assessments. The details of the methodology have been described elsewhere [16].

### Oral health studies in the 1993 Pelotas Birth cohort at ages 6 and 12 yr

The first Oral Health Study (OHS-6) started in December 1998 as a cross-sectional study nested in the birth cohort. In 1998, a sample of the original cohort, consisting of all low birth-weight children along with a random of 20% of the remainder, was revisited. Among the 1,460 eligible children, 87% (1,270 children) were located. A sub-sample drawn from this group was examined to estimate the prevalence of dental caries [17], anterior open bite [18], and posterior cross bite [19]. A sample size of 302 was enough to detect a relative risk of at least 1.3 with 80% power, for a caries prevalence of 65% among the non-exposed, and an error type I of 5%. In the same study, we tested whether breastfeeding acted as a protective factor against the development of malocclusion at age 6 yr [19]. The sample size required to test the association between breastfeeding and malocclusion was estimated for an exposure defined as the duration of breastfeeding of <9 months. Considering the detection of relative risks of at least 1.9 for anterior open bite and 2.5 for posterior cross bite, with a prevalence of 54% and 20%, respectively, in children breastfed for <9 months (exposed), a sample of 342 children was needed to provide 80% power at a significance level of 5%. The sample was inflated by 10% to allow for losses or refusals, resulting in a rounded value of 400 children.

As all of the low-birth-weight children were included in the follow-up at 6 and 12 months of age and at 4 yr of age, they were equally over-represented in the OHS-6 (29.7% when compared with 10% in the original cohort). All the analyses were carried out using weights in other to keep each group proportional to their prevalence in the original sample. The weights used were 0.34 (0.10/0.297) for low birth-weight children and 1.27(0.9/0.703) for the rest. A pilot study involving 40 age-matched children was carried out prior to the fieldwork. All the dental examinations were performed at the child's home by three dentists, responsible for the oral examination, and three interviewers, who administered the questionnaires. The parents were informed about the objectives of the study and consent for interview and examinations were obtained.

Examiner calibration exercises were carried out twice in December 1998 and May 1999. One of the authors was the standard examiner (MAP). Intra- and inter-examiner agreement was high, and the values for the measures of agreement calculated on a tooth-by-tooth basis [20] were high in the first and second calibration (minimum κ values were 0.81 and 0.75, respectively). The World Health Organization [21] criteria were used for diagnosing the dental caries. In addition, oral mucosa lesions and the occlusion [22] were also examined.

The independent variables included child's sex, social and economic conditions, oral behaviors, use of dental services, among others. The response rate was 89.7% (*n *= 359), and non-responses were mainly owing to families moving out of the city.

All the 359 children who participated in the OHS-6 were visited in their homes in 2005, when the adolescents were 12 yr-old. Before the beginning of the study, a specially trained secretary contacted all the families, and authorization was obtained prior to the interviews and oral examinations. A structured interview including questions about dental services utilization (time since the last visit, type of dental services), dental pain (in the last month and their severity), and oral behaviors (toothbrushing, flossing, topical fluorides utilization) were applied. In addition, a short version of the *OIDP *[23] was also administered.

The dental examinations started with the fluorosis diagnosis (WHO 1997), followed by dental trauma [24] and associated treatments needs, dental caries diagnosis [21], and gingival bleeding (all the teeth were probed in six sites, and then bleeding was considered after 10 s). In addition, the criteria of the dental aesthetic index (DAI) were adopted for the analysis of specific types of malocclusions and the normative need for orthodontic treatment [21]. Headlamps were used to improve visualization. Each examiner was adequately dressed, and all dental mirrors and CPI probes were previously sterilized.

The questionnaire used was fully tested including the OIDP questions, and a pilot study was carried out with 40 age-matched adolescents who did not participate in the main study. The fieldwork team comprised four pairs of examiners and interviewers. A PhD dental student was the supervisor of the fieldwork team under the orientation of the study coordinators. The calibration was performed on a tooth-by-tooth basis among 40 adolescents aged 11-13 yr enrolled in public and private schools, following the methodology previously described [20]. The examiner reliability was measured using simple and weighted κ statistics (categorical variables) and intra-class correlation coefficients (numeric variables). The minimum value was κ = 0.60 for gingival bleeding, while the vast majority of values were 1.0. A manual with detailed instructions about each aspect of the study was developed and used by the research team during the data collection.

Each home visit ranged between 30 and 40 min. Before leaving the adolescents' house, the interviewer checked the questionnaire. A dental kit with a toothbrush, fluoride toothpaste, and dental floss was given to the adolescent after the visit. The fieldwork supervisor ensured data quality by contacting 10% of the sample by telephone.

A participant was considered lost after four unsuccessful home visits, including at least one at the weekend and one at night. Families who moved out to places no further than 300 kilometers from Pelotas were contacted and invited to participate, to reduce losses. The fieldwork was performed from April to June 2005.

### Outcome variable

The OIDP was used to assess the adolescent's oral health-related impacts on daily life. The OIDP scale (0-9) is an indicator developed to measure the oral impacts that seriously affect the individual's daily life. The OIDP consists of nine items that cover the physical, psychological, and social dimensions of daily living: eating, smiling, studying, speaking, playing sports, mouth cleaning, sleeping, emotion, and social contact. The adolescents were asked if they had an impact on the nine dimensions of their daily life caused by their mouth or teeth. Each of the nine categories was a binary variable (yes/no). Simple count scores were created by adding the nine dummy variables. We analyzed OIDP as a discrete variable ranged from zero to 9.

### Independent variables

The explanatory variables comprised the socioeconomic and demographic characteristics at birth, such as family income (>6, 1.1-6, ≤ 1 Brazilian Minimum Wage), maternal schooling (≥ 9, 5-8, or ≤ 4 yr), maternal employment status at child aged 6 months (no, yes), adolescent skin color (white, black), sex, and family economic status when the child is 12 yr old (A+B, C, and D+E, ANEP - Brazil Criterion for Economic Classification). In addition, the dental status investigated at 6 yr of age as dental caries measured by the dmft index [21], presence of open bite, and cross bite [22], and at the age of 12 yr as dental caries through the DMFT [21], episode of dental pain (last month before the interview), presence of dental trauma [24], fluorosis [21], gingival bleeding (% of the number of teeth), and the Dental Aesthetic Index -DAI [21] components were also included in the analyses. Finally, we considered the use of dental service at the age of 12 yr in the last yr before the interview (routine visit for check-up, treatment, did not attend), and experienced orthodontic treatment until the age of 12 yr (yes/no).

### Statistical analyses

The analyses were performed using STATA 9.0. These included simple sample distribution, sample distribution according to OIDP level and explanatory variables categories. As the OIDP (outcome) was an extent score, the Poisson regression models with robust variance were performed allowing rate ratio estimates.

**Figure 1:**
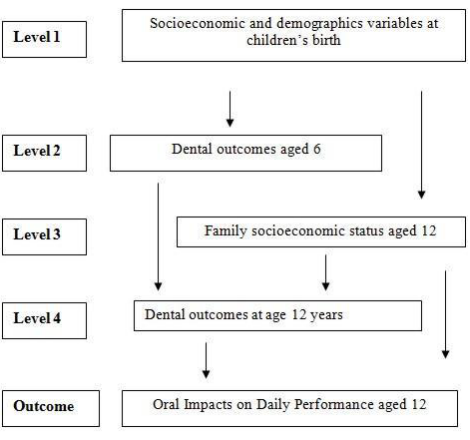
**Conceptual framework of the relationship between life course socioeconomic, demographic and dental status and Oral Impacts on Daily Performance (OIDP)**.

To analyze the potential predictor factors for OIDP, a hierarchical approach to variable selection was used in the multivariate analyses. The independent variables were introduced according to predetermined causality levels from distal to proximate determinants. The choice of variables was based on a conceptual framework describing the hierarchical relationships between the predictor factors [25]. The first level included the socioeconomic variables at birth (maternal schooling, family income, and mother employment status at children age 6 months), sex, and skin color of cohort's participants. The second level included the dental status at the age of 6 yr. The third level comprised the family economic level at 12 yr, and the fourth level added the dental status and use of dental services and orthodontic treatment at 12 yr of age (Figure 1). Complete data on all the factors were not available for all the adolescents. Variables of the first level with *p *value equal or less than 0.25 were retained in the model, and those of the second level were added to it; the second-level variables with *p *> 0.25 were excluded. Finally, variables of the third and fourth levels were included according to the same criterion. The high cutoff was used to ensure that potential confounders were kept in the model. In the final model, the variables were considered as significant if the *p *value was below 0.05, after adjusting for variables in the same level and above, or was retained according to the theoretical framework. Interactions between the dental status retained in the final model were tested using the Wald test for heterogeneity.

Consent for interviews and exams were obtained, and both the projects (at the ages of 6 and 12 yr) were approved by the Pelotas Federal University Ethics Committee. Adolescents who presented dental-treatment needs were referred to the Dental Clinic of the Post-Graduate Program in Dentistry of Pelotas Federal University.

## Results

A total of 339 adolescents were investigated in 2005, representing 94.4% of those investigated in 1999. Around a half of the adolescents were male (53.7%) and one fifth were blacks (20.3%). Adolescent's mother schooling was between 5 and 8 years in the majority of the sample (48.5%), and approximately one third of the mothers worked when the child was 6 months (Table 1).

**Table 1:** Sample distribution of sociodemographic and dental status from birth to 6 yr of age according to OIDP levels (n, %) in adolescents (n = 339) age 12 yr.

Variables	Sample distribution	OIDP = 0	OIDP = 1	OIDP ≥ 2
		
	n (%)	n (%)		
**Sex**				
Male	182(53.7)	76(41.8)	54(29.7)	52(28.5)
Female	157(46.3)	66(42.0)	48(30.6)	43(27.4)
**Skin color**				
White	270(79.7)	113(41.9)	83(30.7)	74(27.4)
Blacks	69(20.3)	29(42.1)	19(27.5)	21(30.4)
Family income at child birth*				
> 6	45(13.3)	21(46.6)	17(37.9)	7(15.5)
1.1-6	232(68.6)	94(40.5)	66(28.5)	72(31.0)
≤ 1	61(18.1)	27(44.3)	18(29.5)	16(26.2)
**Maternal schooling at child birth**				
**≥ 9 yr**	**78(23.1)**	**32(41.0)**	**27(34.6)**	**19(24.4)**
**5 - 8 yr**	**164(48.5)**	**76(46.3)**	**42(25.6)**	**46(28.1)**
**≤ 4**	**96(28.4)**	**34(35.4)**	**32(33.3)**	**30(31.3)**
**Mother employment status at child aged 6 month**				
**No**	**230(68.1)**	**110(47.8)**	**67(29.1)**	**53(23.1)**
**Yes**	**108(31.9)**	**32(29.6)**	**34(31.5)**	**42(38.9)**
**Untreated dental caries at age 6**				
0	124(36.7)	54(43.5)	40(32.3)	30(24.2)
1-3	92(27.2)	38(41.3)	25(27.2)	29(31.5)
4-19	122(36.1)	50(41.0)	36(29.5)	36(29.5)
**Open bite at age 6 yr**				
No	173(52.2)	73(42.2)	49(28.3)	51(29.5)
Yes	165(48.8)	69(41.8)	52(31.5)	44(26.7)
**Cross bite at age 6 yr**				
No	277(81.9)	120(43.3)	85(30.7)	72(26.0)
Yes	61(18.1)	22(36.1)	16(26.2)	23(37.7)

Pelotas, Brazil, 2005.

*BMW = Brazilian Minimum Wage (around US$ 190,00 in June 2007)

**Figure 2:**
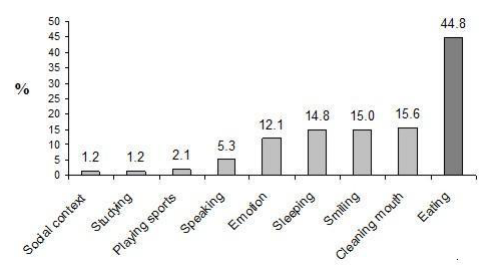
**Prevalence of each oral health impact on daily performances on adolescents age 12 yr**. Pelotas, Brazil, 2005.

Almost 50% of the adolescents belonged to the two lower family economic categories according to the Brazilian socioeconomic classification. Dental pain affected 12.1% of the adolescents and similar prevalence of dental trauma (14.9%) and dental fluorosis (14.9%) were also observed. The highest prevalence of malocclusion identified was related to anterior segment spacing in adolescents (39.2%). The percentage of adolescents who never visited a dentist was 66.3%, and almost all of them were never submitted to orthodontic treatment (93.2%). The prevalence of no impact (OIDP = 0) was 41.9% (95%CI 36.6; 47.2), while OIDP = 1 achieved 30.1% (95%CI 25.2; 35.0), and OIDP ≥ 2 affected 28.0% (95%CI 23.2; 32.8) of the sample (Table 2). The most common daily performance affected at age 12 yr was eating (44.8%), followed by cleaning of the mouth, and smiling (15.6% and 15.0%, respectively) (Figure 2).

**Table 2:** Sample distribution of current socioeconomic, dental status, and dental visit according to OIDP levels (n, %) in adolescents (n = 339) age 12 yr.

Variables	Sample distribution	OIDP = 0	OIDP = 1	OIDP ≥ 2
		
	n (%)	n (%)		
Family economic status at age 12 **				
A + B	63(18.9)	30(47.6)	16(25.4)	17(27.0)
C	108(32.3)	49(45.4)	35(32.4)	24(22.2)
D + E	163(48.8)	61(37.4)	48(29.5)	54(33.1)
**Untreated dental caries at age 12**				
No	200(59.0)	96(48.0)	64(32.0)	40(20.0)
Yes	139(41.0)	46(33.1)	38(27.3)	55(39.6)
**Dental pain at age 12**				
No	298(87.9)	134(44.9)	92(30.9)	72(24.2)
Yes	41(12.1)	8(19.5)	10(24.4)	23(56.1)
**Dental trauma at age 12**				
No	285(85.1)	119(41.8)	85(29.8)	81(28.4)
Yes	50(14.9)	23(44.2)	15(28.9)	14(26.9)
**Dental fluorosis at age 12**				
No	285(85.1)	115(40.1)	87(30.5)	83(29.1)
Yes	50(14.9)	24(48.0)	15(30.0)	11(22.0)
**Gingival bleeding at age 12 (% teeth affected)**				
<11.5	113(33.3)	53(46.9)	40(35.4)	20(17.7)
11.5-28.0	110(32.5)	51(46.4)	28(25.5)	31(28.1)
28.5-92.0	116(34.2)	38(32.8)	34(29.3)	44(37.9)
**Incisal crowding at age 12**				
No	253(74.6)	111(43.9)	83(32.8)	59(23.3)
Yes	86(25.4)	31(36.0)	19(22.1)	36(41.9)
**Maxillary anterior crowding at age 12**				
No	229(67.6)	101(44.1)	66(28.8)	62(27.1)
Yes	110(32.4)	41(37.3)	36(32.7)	33(30.0)
**Mandible anterior crowding at age 12**				
No	261(76.9)	111(42.5)	80(30.7)	70(26.8)
Yes	78(23.1)	31(39.7)	22(28.2)	25(32.1)
**Anterior segment spacing at age 12**				
No	206(60.8)	94(45.6)	53(25.7)	59(28.6)
Yes	133(39.2)	48(36.1)	49(36.8)	36(27.1)
**Maxillary overjet at age 12**				
≤ 3 mm	245(73.3)	109(44.5)	71(29.0)	65(26.5)
> 3 mm	94(27.7)	33(35.1)	31(33.0)	30(31.9)
**Anterior open bite at age 12**				
No	314(92.6)	131(41.7)	93(29.6)	90(28.7)
Yes	25(7.4)	11(44.0)	9(36.0)	5(20.0)
**Dental visit at age 12**				
Routine visit	47(13.9)	21(44.6)	13(27.7)	13(27.7)
Treatment	67(19.8)	27(40.2)	20(29.9)	20(29.9)
Did not attend	224(66.3)	94(42.0)	68(30.3)	62(27.7)
**Orthodontic treatment until age 12**				
Yes	23(6.8)	10(43.5)	6(26.1)	7(30.4)
No	316(93.2)	132(41.8)	96(30.4)	88(27.8)

**Total**	339	142(41.9)	102(30.1)	95(28.0)

Pelotas, Brazil, 2005.

**According to the Brazil Criterion for Economic Classification proposed by ANEP.

Table 3 shows the unadjusted and adjusted rate ratio from Poisson multivariable regression analysis for the association between OIDP score and demographic, socioeconomic and dental status variables. Among the variables belonging to the first level (demographic and socioeconomic during the early life), maternal schooling at child birth and maternal employment status when children was 6 months remained associated with the outcome after adjustment. As lowest adolescent's mother schooling as highest the OIDP score. Adolescents whose mother had worked at child birth showed highest OIDP score compared with their counterparts. In the level 2 (dental status at aged 6), as higher the number of untreated dental caries as higher the OIDP score. The presence of crossbite was also associated with higher OIDP score after adjusted for the variables in the model. Finally, in the most proximal level (dental status, dental visit, and current socioeconomic at aged 12) it was observed that adolescents presenting untreated dental caries, dental pain, severe gingival bleeding, and incisal crowding, showed higher OIDP score when compared with those free of these conditions. In addition, the presence of dental fluorosis showed a negative association with OIDP score.

**Table 3:** Simple and multiple Poisson regression analysis of the relationship between socio-demographic and dental status variables according to OIDP (as discrete variable) in adolescents age 12 yr.

Variables	Unadjusted Rate Ratio (IC 95%)	P	Adjusted Rate Ratio (IC 95%)	P
	
Level 1				
Sex		0.170		0.104^a^
Male	1.0		1.0	
Female	1.2 (0.9;1.4)		1.2 (1.0;1.5)	
Maternal schooling at child birth		0.141		0.013^a^
≥ 9 yr	1.0		1.0	
5 - 8 yr	1.1 (0.8;1.4)		1.2 (0.9;1.6)	
≤ 4	1.2 (0.9;1.6)		1.4 (1.0;1.9)	
Mother employment status at child aged 6 month		<0.001		<0.001^a^
No	1.0		1.0	
Yes	1.5 (1.2;1.9)		1.6 (1.3;2.0)	

**Level 2**				

Untreated dental caries at age 6		0.043		0.016^b^
0	1.0		1.0	
1-3	1.2 (1.0;1.6)		1.2 (1.0;1.6)	
4-19	1.3 (1.0;1.6)		1.4 (1.1;1.7)	
Cross bite at age 6 yr		0.020		0.058^b^
No	1.0		1.0	
Yes	1.3 (1.0;1.7)		1.3 (1.0;1.6)	

**Level 3**				

Family economic status at age 12		0.138		0.612^c^
A + B	1.0		1.0	
C	1.1 (0.8;1.4)		1.0 (0.7;1.4)	
D+E	1.2 (0.9;1.6)		1.1 (0.8;1.5)	

**Level 4**				

Untreated dental caries at age 12		<0.001		0.029^d^
No	1.0		1.0	
Yes	1.6 (1.3;1.9)		1.3 (1.0;1.6)	
Dental pain at age 12		<0.001		<0.001^d^
No	1.0		1.0	
Yes	2.2 (1.8;2.8)		1.9 (1.5;2.5)	
Dental fluorosis at age 12		0.120		0.046^d^
No	1.0		1.0	
Yes	0.8 (0.6;1.1)		0.7 (0.5;1.0)	
Gingival bleeding at aged 12		0.004		0.047^d^
<11.5% of teeth	1.0		1.0	
11.5-28.0% of teeth	1.3 (1.0.1.7)		1.1 (0.9;1.5)	
28.5-92.0% of teeth	1.5 (1.1;1.9)		1.3 (1.0;1.7)	
Incisal crowding at aged 12		<0.001		0.003^d^
No	1.0		1.0	
Yes	1.5 (1.2;1.9)		1.4 (1.1;1.8)	

Pelotas, Brazil, 2005 (*n *= 339).

Level 2: adjusted by variables from level 1

Level 4: adjusted by variables from level 1 e and level 2

## Discussion

This study investigated the prevalence of the impact of dental status on the day-to-day life in a population-based birth cohort of 12-yr-old adolescents from Pelotas in Southern Brazil, using a life-course approach. A positive association between the cohort partticipant's mother level of education, mother employment status at child early life, beyond the dental status during the life and OIDP was found.

The prevalence of at least one oral impact experienced during the past 6 months by the studied population was high (58.1%), while 28.0% of the cohort participants had two or more impacts. Similar findings for at least one impact were reported among schoolchildren from Uganda (62%) [5], but not among British adolescents, where the prevalence was only 26.5% [8]. Previous studies carried out in different Brazilian cities found the prevalence of 27.5% and 32.8% [6,9] of at least one impact.

In our study, the most common daily performances affected by oral health conditions were eating, cleaning of the mouth, and smiling. Eating was also the most frequently affected daily performance observed in Uganda [5], but executing oral hygiene and smiling was observed to be the main causes of impact in a small town in South Brazil [26] and London [8]. The aforementioned studies investigated older adolescents than those investigated in this study, and the range of age differences may explain the different results. On the other hand, the epidemiological figures of oral diseases can significantly influence the pattern of the causes of such impacts. For example, early dental pain affected 12.1% and untreated dental caries affected almost half (41.0%) of the adolescents. Therefore, it is understandable that eating have been self-reported as the main impact, corroborating other study developed in Thailand [12].

It is important to mention that during the protocol development of the oral health study in the Pelotas cohort, the Child-OIDP version [27] was not yet validated in Brazilian Portuguese. Hence, we used the general OIDP [23] that was previously validated in a sample of Brazilian adolescents [28]. Studies that investigated the oral health-related quality of life through Child-OIDP index showed the prevalence of overall impact ranging from 15.5% among 11-12-yr-old Peruvian schoolchildren [29] to 28.6% of Tanzanian schoolchildren aged 12-14 yr [30], which is much lower than our findings, or on the other hand, much higher (89.8%) than that found in Thai schoolchildren [12].

Among socioeconomic and demographic variables investigated only those related to the cohort participants mothers - schooling and work status in child early life - were associated with OIDP in adolescence. Level of education is an important marker of socioeconomic position; higher education level generally is predictive of better jobs, higher incomes and better housing and socio-economic position [31]. Consequently, mother's level of education is one of the best predictors for children health, especially in developing countries [32]. In the field of oral health, it is very known that maternal cognitive, behavioral, and psychosocial factors are associated with children oral behaviours as, for example, toothbrushing [33].

There is a lack of studies addressing the relationship between maternal work, maternal employment status and child oral health. On the other hand, findings from the UK Millennium Cohort Study showed that children whose mothers worked were more likely to primarily drink sweetened beverages between meals, they were likely to eat fruit/vegetables between meals compared to other snacks [34]. The pattern of sugar consumption is strongly associated with dental caries, dental pain and, consequently, impacts on daily life.

Untreated dental caries in both deciduous and permanent dentition was associated with OIDP in adolescence. Dental pain at the age of 12 yr was also strongly associated with OIDP levels, corroborating with another study that showed care-seeking being associated with dental pain, difficulties in sleeping, and difficulties in playing among adolescents [10,11]. Dental pain in adolescence is a dental public-health concern in Brazil [15] and worldwide [11,35], and its assessment can add to the best knowledge of dental-need estimation to achieve one of the Global Goals for Oral Health 2020 [36]. As expected, dental fluorosis was associated with low OIDP score. Having mild fluorosis was significant factor for adolescent's perception of good global rating of oral health [37].

The impact of malocclusion and orthodontic-treatment needs on OIDP has been deeply investigated [6-9,29]. In most of these studies, poor oral health-related quality of life were shown in adolescents with self-perceived malocclusion [29], as well as in those presenting normative orthodontic treatment needs [8]. Hypothetically, malocclusions might have a strong influence on activities, such as smiling, emotion, and social contact. Our results confirm that dentofacial aesthetics play an important role in social interactions and psychosocial well-being. However, it was restricted to incisal crowding, which was also demonstrated in another research [9]. Unlike the other studies, we statistically controlled the impact of different occlusal traits on the OIDP by early life socioeconomic and demographic variables, as well as by the most important oral outcomes.

No difference in the OIDP was found between boys and girls, probably because at this early phase of adolescence, gender-related behaviors are not prominent. We presume that in the subsequent assessment of this cohort in the late adolescence, the differences between boys and girls in health-related quality of life and satisfaction will be revealed, as in another study [38]. Previous studies have shown consistent differences between young males and females in their dental behaviours and pattern of dental attendance, with women generally having more favourable behaviours than men. These gender differences may influence dental status later in life and then, consequently impact of oral health on daily life [39].

Some important psychosocial variables that possibly act during childhood were not collected in our study. Further studies need to be developed to clarify the complex relationship between social and psychological factors.

Some additional commentaries about the study methods are relevant. The sample investigated at the age of 12 yr did not differ significantly from the original cohort and the 6-yr-old sample. For example, proportion of males (53.9 vs. 53.7%) and family income equal to or lesser than the Brazilian Minimum Wage per month (17.8% vs. 18.1%) observed at 6 and 12 yr of age, respectively, suggest the lack of attrition bias [17]. In addition, high levels of diagnostic reliabilities, the use of blinded examiners/interviewers, knowledge of the prospective factors investigated, as well as a population-based design contribute to the strengths of the study. Measures of oral health-related quality of life have been largely incorporated in oral health surveys to improve the assessment of perceived need and the impact of the outcomes of dental care. In our study, some major methodological improvements were achieved in comparison with the previous reports. First, we analyzed several oral conditions at the same time, including various individual occlusal traits. Second, the simultaneous evaluation of several oral conditions rather than assessing specific outcome was possible with an overview of the dental health needs as well, and consequently, it allowed the prioritization of services planning. Third, it enabled us to verify the impact of early life oral conditions in the adolescent oral health-related quality of life owing to a longitudinal study design. Finally, the use of Poisson regression models instead ordinary logistic regression allowed complete utilization of original OIDP, a ranked data.

The main methodological limitation of the study is the use of general OIDP questionnaire that had been developed for use in adult populations [23], as the Child-OIDP questionnaire had not been previously validated in Brazil [27]. Moreover, the lack of incidence measures and the need for a larger sample to enhance statistical power are the other limitations of our study.

In conclusion, oral impact on adolescents' day-to-day life was a common finding in our study. We highlighted the importance of adolescent's early life social environmental as mother schooling and mother employment status and dental status that may cause suffering, such as untreated dental caries in both deciduous and permanent dentition, gingival bleeding, and dental pain, besides malocclusion, which is an aesthetical problem. Competing interestsThe authors declare that they have no conflict of interests.

## Authors' contributions

KGP conceived the study, performed the statistical analysis and interpretation of data, and drafted the manuscript. MAP participated in the collection, analysis and interpretation of data, and revising critically the manuscript. CLPA, AMBM, and PCH helped the interpretation of data and revising critically the manuscript. All authors read and approved the final version of the manuscript.

## Acknowledgements

Karen Glazer Peres, Marco Aurélio Peres, Ana MB Menezes, and Pedro Curi Hallal received grants for productivity in research from the CNPq (Conselho Nacional de Desenvolvimento Científico e Tecnológico). The cohort study is supported by the Wellcome Trust. The initial phases of the cohort study were financed by the European Union, by the PRONEX (Programa de Apoio a Núcleos de Excelência), by the CNPq, and by the Brazilian Ministry of Health.
